# Preparedness for diphtheria epidemic among healthcare workers in Kano State, Nigeria

**DOI:** 10.12688/openreseurope.22188.1

**Published:** 2026-04-13

**Authors:** Ayodele Arinola Feyisara, Noah David, Etim Utibe Efre, Kevin Dofwah Nuhu, Azibadighi Walter, Olufunmilayo Ibitola Fawole, Adeniyi Francis Fagbamigbe, Segun Bello, Onoja Matthew Akpa

**Affiliations:** 1 Epidemiology and Medical Statistics, University of Ibadan Faculty of Public Health, Ibadan, Oyo, Nigeria; 2 Epidemiology and Community Health, University of Ilorin Teaching Hospital, Ilorin, Kwara, Nigeria; 3 Fellow, European and Developing Countries Training Program (EDCTP) MPH EBDOER, Ibadan, Nigeria; 4 Pathology, Aminu Kano Teaching Hospital, Kano, Kano, Nigeria; 5 Veterinary and Pest Control Services, Ministry of Agriculture, Uyo, Akwa Ibom, Nigeria; 6 Department of Epidemiology and Medical statistics, College of Medicine, University of Ibadan, Ibadan, Oyo, Nigeria

**Keywords:** Diphtheria, healthcare workers, preparedness

## Abstract

**Background:**

Ensuring epidemic preparedness is a critical responsibility of every sovereign nation. Every threat to health, particularly the spread of communicable diseases, constitutes a potential threat to human existence. Healthcare Workers (HCWs) are the first line of defense against disease outbreaks; however, their knowledge levels vary. This study aimed to assess the knowledge of and preparedness for the diphtheria epidemic among healthcare workers in Kano state, Nigeria, using a quantitative method.

**Methods:**

This study used a descriptive, cross-sectional design. A two-stage sampling technique was used to select 421 HCWs, who responded to a semi-structured interviewer-administered questionnaire. Descriptive statistics and logistic regression were used for the analysis at the 5% significance level.

**Results:**

Results showed that 51% of healthcare workers had poor knowledge of diphtheria and 53.4% demonstrated generally poor infection prevention control (IPC) practices. Factors such as age group, education level, ethnicity, and facility level were associated with knowledge and IPC practices of diphtheria. The sociodemographic predictors of knowledge of diphtheria were age group (OR = 1.320, CI 0.669–2.606, p-value 0.424). Facility level predicted IPC practices, those in primary health facilities were 91% less likely to have good IPC practices compared to those in tertiary facilities, with statistically significant finding of (OR = 0.097, CI 0.041–0.232, p-value 0.001).

**Conclusions:**

Overall, 78.1% of respondents demonstrated good preparedness for diphtheria outbreaks. However, facility preparedness for diphtheria response was found to be lacking, with 70.8% of facilities having poor preparations. Preparedness is vital for effective disease outbreak responses, necessitating collective efforts for robust future responses.

## Plain Language summary

This study looks at how ready healthcare workers are to prevent and respond to diphtheria, a serious but preventable infectious disease, in Kano State, Nigeria. Diphtheria spreads easily from person to person and can be deadly, especially for children, if it is not detected and treated early. Because doctors, nurses, and other health workers are the first people to see and treat patients during outbreaks, their knowledge, safety practices, and readiness are very important.

The researchers asked over 400 healthcare workers questions about diphtheria—how it spreads, how to protect themselves and patients, and how prepared their workplaces are to handle an outbreak. The findings showed that about half of the health workers did not have good knowledge of diphtheria, and more than half did not consistently follow good infection prevention and control practices, such as proper use of protective equipment and hygiene measures. Although many individual health workers felt personally prepared to respond to an outbreak, most health facilities lacked important supplies, plans, and systems needed for a strong response.

The study highlights a clear message: improving training, supplies, and preparedness in health facilities—especially in primary healthcare centers—will better protect health workers, patients, and communities, and help stop future diphtheria outbreaks more effectively.

**List of abbreviations**
HCWs: Healthcare Workers
IPC: Infection Prevention Control
IHR: International Health Regulations
IDSR: Integrated Disease Surveillance and Response
SPSS: Statistical Package for the Social Sciences

## Introduction

Diseases have no limits; a health threat anywhere is a health threat everywhere. Recent instances of disease outbreaks underscore the interconnected nature of global health, emphasizing that a threat posed by a disease in any location poses a risk to the entire world ( CDC, 2023a). Emergence and new infectious diseases are today’s top global health security risks, and one way to combat this is by workforce development of frontline staff to identify, track and contain outbreaks at their source. The negative and destructive effects of outbreaks are more likely to occur in a world where there is no constant focus on ensuring global health security. The goal of the global health security agenda is to improve the global capacity to prevent, identify, and address infectious disease threats. Nigeria is among the countries that have signed this framework. In 2019, Nigeria became a global health security agenda partner country committed to improving progress towards global health security agenda goals and internal health regulations ( CDC, 2023b).

Preparation is a requirement of 196 signatories to the International Health Regulations (IHR) (2005), the world’s defense against significant threats to public health. Systems must be capable of preventing, defending against, controlling, and responding to the international spread of illness, which can occur at unanticipated rates in an interconnected world ( WHO, n.d.). Epidemic preparedness refers to all the steps that are needed to be performed at the local, state, and federal levels, as well as in health facilities to be prepared and successfully address disease outbreaks. To prepare for an epidemic, routine surveillance systems must be developed to detect outbreaks, and employees must be organized to confirm, examine, and respond to epidemics ( Fatiregun and Isere, 2017). To deal with the disease, it is important to prepare a culture of safe medical procedures that can limit and stop the spread of disease outbreaks. Without a culture of safe practices, healthcare facilities will be more vulnerable to communicable disease exposure and health disruption ( Ughasoro et al., 2019).

Diphtheria, a vaccine-preventable disease and an epidemic-prone notifiable disease reported under the integrated disease surveillance and response system (IDSR), has been a global burden that has improved with the introduction of vaccines into immunization programs. However, in Nigeria today, there is a surge in the incidence of this contagious disease, causing epidemics that primarily affect children. It is caused by Corynebacterium spp., mainly by toxin-producing *Corynebacterium diphtheriae* and rarely by toxin-producing strains of *Corynebacterium ulcerans* and *Corynebacterium pseudotuberculosis.* It appears as tonsillitis, laryngitis, or pharyngitis with signs and symptoms such as fever, cough, running nose, sore throat, and red eyes, and is characterized by an adherent pseudo-membrane, a thick, grey coating made up of necrotic tissue and bacteria, which is the tell-tale sign of infection covering the tonsils, pharynx, and/or nose. In addition to respiratory issues, myocarditis and neuritis may develop in some cases. The low vaccine coverage of diphtheria antitoxin has been attributed to disease outbreaks. As of November 2023, Epi week 01–44 cumulatively, 20 states have been affected, 130 LGAs, a total of 10,863 have been confirmed cases, 560 deaths, and a case fatality of 5.2% has been reported. The immunization coverage rate ranged from 5–48% across northern states in 2018, of which Kano state is one of them ( Eze et al., 2021). Kano State is in the northwestern part of the country, is one of the states currently hit by diphtheria, and has the highest number of cases and deaths. Skilled human resources are crucial components required to successfully respond to an infectious disease outbreak. This review aims to assess preparedness for the diphtheria epidemic among health workers in Kano State, Nigeria.

## Methodology

This descriptive cross-sectional study was designed to assess the preparedness for the diphtheria epidemic among healthcare workers in the Kano state using a quantitative study approach to collect data from 421 healthcare workers (Nurses and Community Health Extension workers) through a semi-structured interviewer questionnaire.

A multistage sampling technique was used for Healthcare Workers (HCWs), and a semi-structured interviewer-administered questionnaire was used to obtain quantitative data. Data generated from the study were analyzed using Statistical Package for the Social Sciences (IBM/SPSS version 21). The sex variable used in this study applies to the sex assigned at birth.

### Ethical approval statement and consent to participate

Ethical approval was obtained from the Health Research Ethics Committee of the Kano State Ministry of Health, Kano State, Nigeria on the 19 ^th^ of May 2023.The approval number is NHREC/17/03/2018 and the reference number is SHREC/2023/3985.The study commenced in September 2023 where written informed consent was obtained from all the participants. No harm was caused to the participants during the study Participants were informed about the choice to participate or withdraw from the study at any time with no consequences.

***Consent to participate***

A written informed consent was obtained from all participants, and this document was approved by the ethics committee before the study was implemented.

## Results

The mean (standard deviation) age of the health workers who participated in the study was 34(9.2%) years, indicating that they predominantly fell within the early to mid-thirties age range. The majority (54.7%) of the participants were female, while (45.8%) were male. The predominant religion observed was Islam (80.5%), whereas (19.5%) are Christians. Regarding educational background, 47.7% had a certificate or diploma,8.3% had an undergraduate degree, and 43.9% had a graduate or postgraduate degree. The ethnic composition is diverse, with the largest proportion being Hausa (58.9%) and the majority (72.5%) of the respondents being married. Almost half of the respondents 46.1% are community health extension workers, while slightly above half 53.9% are nurses. There was a fair distribution of respondents across the senatorial districts, with 47.0% from the primary health care institutions ( Table 1).

**Table 1.** Socio-demographic characteristics of the respondents.

Socio-demographics	Frequency
**Age group (years)**	
<=25	83 (19.7)
26–30	113 (26.8)
31–35	77 (18.3)
36–40	62 (14.7)
41+	86 (20.5)
Mean age ± SD (years)	34 ± 9.2
**Sex**	
Female	228 (54.2)
Male	193 (45.8)
**Highest level of education**	
Certificate/diploma	201 (47.7)
Undergraduate	35 (8.3)
Graduate/postgraduate	185 (44.0)
**Religion**	
Islam	339 (80.5)
Christianity	82 (19.5)
**Ethnicity**	
Fulani	75 (17.8)
Hausa	248 (58.9)
Igbo	55 (13.1)
Yoruba	35 (8.3)
Others	8 (1.9)
**Marital status**	
Never married	110 (26.1)
Married	305 (72.5)
Divorced/separated/widowed	6 (1.4)
**Cadre**	
Community health worker	194 (46.1)
Nurse	227 (53.9)
**Facility level**	
Primary	198 (47.0)
Secondary	109 (25.9)
Tertiary	114 (27.1)
**Senatorial district**	
Kano South	132 (31.4)
Kano North	139 (33.0)
Kano Central	150 (35.6)

### Overall knowledge grading of respondents

**Figure 1.**
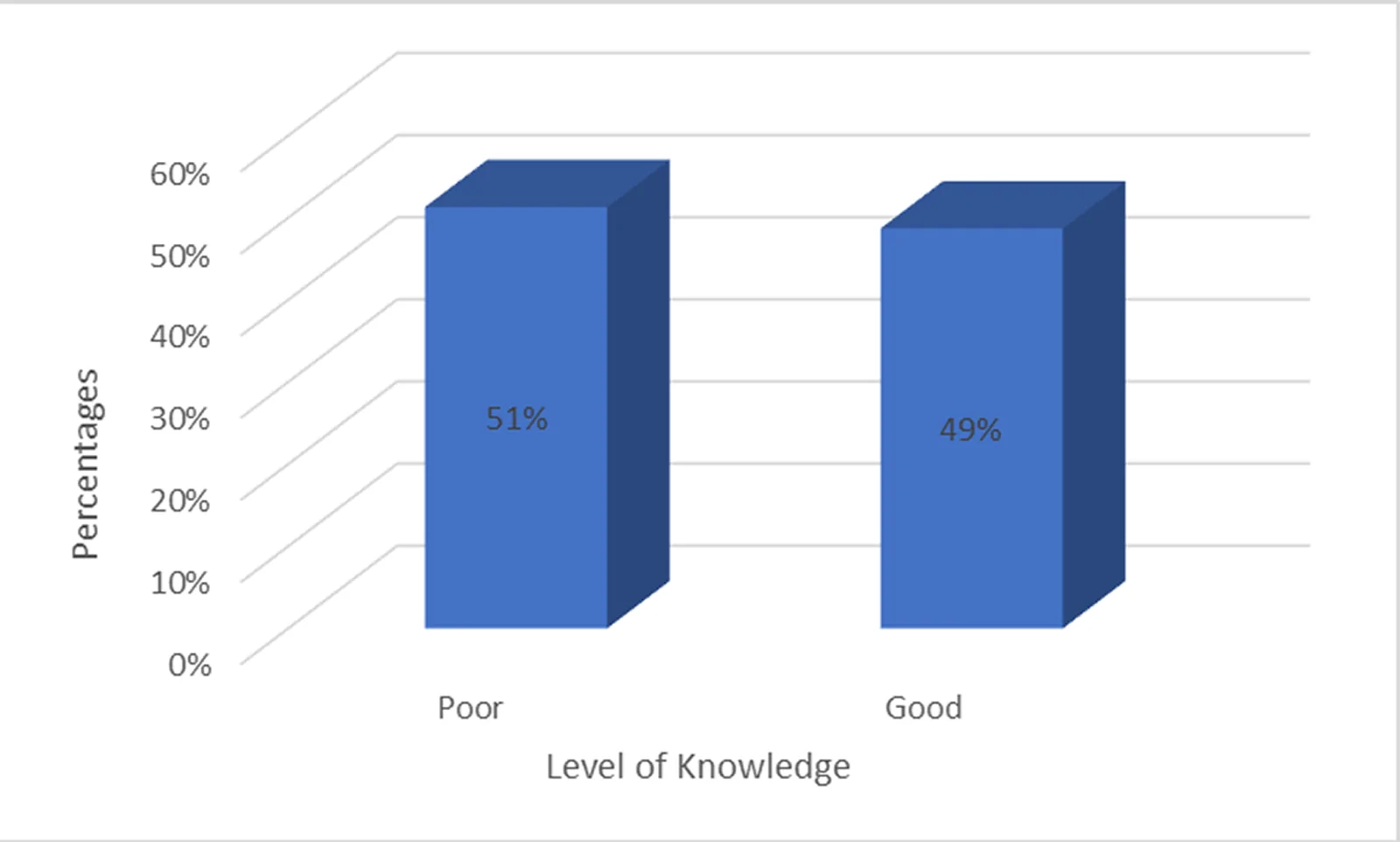
The level of Knowledge among respondents.

For the overall knowledge score, slightly above half of the respondents 51% have poor knowledge as compared with 49% of the respondents who had good knowledge ( Figure 1).

### Respondents’ association between socio-demographic characteristics and knowledge of diphtheria

The distribution of levels (poor or good) of knowledge of diphtheria and the background characteristics of the respondents is shown in Table 2. Notably, respondents of other ethnicities (62.2%) demonstrated a higher proportion of good knowledge compared to the Hausa (44.4%) and the Fulani (50.0%) ethnic groups. Health care workers with a graduate/postgraduate degree have a higher proportion of good knowledge (56.2%) compared to those with a certificate/diploma or undergraduate degree (37.1%). On average, the proportion of healthcare workers with good knowledge increases from younger to older age groups. A statistically significant association was found between age group, highest level of education, ethnicity, and the knowledge of diphtheria, with the p-values of 0.004, 0.018, and 0.018, respectively. Community Health Workers had 55.2% in the poor knowledge category and 44.8% in the good knowledge category, while nurses had 48.0% and 52.0%, respectively, the chi-square analysis for cadre yielded a value of 2.133 with a p-value of 0.144, indicating no significant association. Similarly, there was no significant association at the facility level (chi-square value = 4.278, p = 0.118) or among senatorial districts (chi-square = 2.796, p = 0.247), despite some variability in knowledge distribution at Primary, Secondary, and Tertiary facility levels, as well as among the Kano South, Kano North, and Kano Central districts ( Table 2).

**Table 2.** Association between Socio-demographic characteristics and knowledge of diphtheria among Healthcare Workers.

Parameters	Knowledge			
	Poor (%)	Good (%)	χ ^2^	p-value
**Age group (years)**				
≤ 25	57 (68.7)	26 (31.3)	15.323	0.004*
26–30	50 (44.2)	63 (55.8)		
31–35	42 (54.5)	35 (45.5)		
36–40	26 (41.9)	36 (58.1)		
> 40	41 (47.7)	45 (52.3)		
**Sex**				
Female	114 (50.0)	114 (50.0)	0.340	0.560
Male	102 (52.8)	91 (47.2)		
**Highest level of education**				
Certificate/diploma	113 (56.2)	88 (43.8)	8.001	0.018*
Undergraduate	22 (62.9)	13 (37.1)		
Graduate/postgraduate	81 (43.8)	104 (56.2)		
**Religion**				
Islam	185 (54.6)	154 (45.4)	7.431	0.006*
Christianity	31 (37.8)	51 (62.2)		
**Ethnicity**				
Fulani	38 (50.7)	37 (49.3)	11.907	0.018*
Hausa	141 (56.9)	107 (43.1)		
Igbo	19 (34.5)	36 (85.5)		
Yoruba	16 (45.7)	19 (54.3)		
Others	2 (25.0)	6 (75.0)		
**Marital status**				
Not married	66 (56.9)	50 (43.1)	6.779	
Married	150 (49.2)	155 (50.8)		
**Cadre**				
Community health worker	107 (55.2)	87 (44.8)	2.133	0.144
Nurse	109 (48.0)	118 (52.0)		
**Facility level**				
Primary	91 (46.0)	107 (54.0)		
Secondary	61 (56.0)	48 (44.0)	4.278	0.118
Tertiary	64 (56.1)	50 (43.9)		
**Senatorial district**				
Kano South	62 (47.0)	70 (53.0)	2.796	0.247
Kano North	79 (56.8)	60 (43.2)		
Kano Central	75 (50.0)	75 (50.0)		

### Respondents’ association between socio-demographic characteristics and infection prevention control practices

Overall, the level of infection prevention control practices among the health workers showed that slightly above half (53.4%) of the respondents had poor Infection Prevention Control (IPC) practices, while 46.6% had good practices. There was no significant association between age or sex. However, a significant association existed between the highest level of education and diphtheria practice (χ ^2^ = 12.395, p = 0.002). Specifically, healthcare workers with a certificate/diploma show a higher proportion of poor practice than those with an undergraduate, graduate, or postgraduate degree. No significant association was found between ethnicity and Diphtheria practice (χ ^2^ = 4.018, p = 0.404) There was a notable association between cadre, health facility level, and senatorial district with IPC practice among respondents. Specifically, Community Health Workers, Primary facilities, and the senatorial districts of Kano South and Kano North exhibited higher proportions of poor practices than Nurses, Secondary and Tertiary facilities, and Kano Central facilities, with all associations being highly significant (p = 0.001) ( Table 3).

**Table 3.** Association between socio-demographic characteristics and infection prevention control practice of diphtheria among healthcare workers.

Variables	Practice		χ ^2^	p-value
	Poor (%)	Good (%)		
**Age group (years)**				
≤ 25	47 (56.6)	36 (43.4)	5.762	0.218
26–30	67 (59.3)	46 (40.7)		
31–35	40 (51.9)	37 (48.1)		
36–40	34 (54.8)	28 (45.2)		
> 40	37 (43.0)	49 (57.0)		
**Sex**				
Female	117 (51.3)	111 (48.7)	0.905	0.341
Male	108 (56.0)	85 (44.0)		
**Highest level of education**				
Certificate/diploma	123 (61.2)	79 (38.8)	12.395	0.002*
Undergraduate	21 (60.0)	14 (40.0)		
Graduate/postgraduate	81 (43.8)	104 (56.2)		
**Ethnicity**				
Fulani	41 (54.7)	34 (45.3)	4.018	0.404
Hausa	134 (54.0)	114 (46.0)		
Igbo	32 (58.2)	23 (41.8)		
Yoruba	16 (45.7)	19 (54.3)		
Others	2 (25.0)	6 (75.0)		
**Marital status**				
Never married	55 (50.0)	55 (50.0)	1.829	0.425 ^f^
Married	168 (55.1)	137 (44.9)		
Divorced/separated/widowed	2 (33.3)	4 (66.7)		
**Cadre**				
Community health worker	124 (63.9)	70 (36.1)	15.862	**0.001**
Nurse	101 (44.5)	126 (55.5)		
**Facility level**				
Primary	155 (78.3)	43 (21.7)	108.454	**0.001**
Secondary	49 (45.0)	60 (55.0)		
Tertiary	21 (18.4)	93 (81.6)		
**Senatorial district**				
Kano South	99 (75.0)	33 (25.0)	66.408	**0.001**
Kano North	84 (60.4)	55 (39.6)		
Kano Central	42 (28.0)	108 (72.0)		

### Respondents’ socio-demographic predictors of knowledge of diphtheria

The sociodemographic predictors of knowledge of diphtheria among the health workers showed that age, education level, ethnicity, and marital status did not consistently exhibit statistically significant associations with knowledge of diphtheria ( Table 4).

**Table 4.** Socio-demographic predictors of knowledge of diphtheria among study participants.

Variables	β	Odd ratio	95% C I	p-value
**Age group (years)**				
≤ 25	−0.561	0.570	0.280–1.161	0.121
26–30	0.334	1.397	0.757–2.579	0.285
31–35	−0.143	0.867	0.457–1.645	0.662
36–40	0.278	1.320	0.669–2.606	0.424
> 40	RC			
**Highest level of education**				
Certificate/diploma	0.445	1.561	0.715–3.408	0.264
Undergraduate	0.673	1.960	0.887–4.332	0.096
Graduate/postgraduate	RC			
**Ethnicity**				
Fulani	−0.052	0.949	0.408–2.206	0.903
Hausa	−0.280	0.756	0.357–1.602	0.465
Igbo	0.493	1.637	0.668–4.010	0.281
Others	1.030	2.802	0.474–16.548	0.256
Yoruba	RC			
**Marital status**				
Never married	0.055	1.057	0.650–1.719	0.823
Married	RC			

RC = Reference category.

### Respondents’ predictors of infection prevention control practices

Those working in the primary health facilities were 91% less likely to have good IPC practices against diphtheria as compared to the tertiary health facilities, and this was statistically significant (CI 0.041–0.232, p-value 0.001). Also, those working in the secondary health facilities are 60% less likely to have good IPC practices against diphtheria as compared in the tertiary health facilities. Those with certificate/diploma are 2.4 times more likely to have good preventive practices when compared to Graduate/postgraduate, but this is not statistically significant (CI 0.982–6.090, p-value 0.055). In addition, respondents with undergraduate education, are 2 times more likely to have good preventive practices against diphtheria, and this was also not statistically significant. (CI:0.905–5.072, p-value 0.083). Those who are Community Health Workers are 25% less likely to have good preventive practices as compared with the Nurses (CI 0.452–1.274, p-value 0.297) though not statistically significant. The senatorial district did not demonstrate a statistically significant association with the preventive practice of diphtheria compared with the reference group (Kano central) as shown in ( Table 5).

**Table 5.** Predictors of Infection prevention control practice of diphtheria among study respondents.

Variables	Β	Odd ratio	95% C I	p-value
**Highest level of education**				
Certificate/diploma	0.894	2.445	0.982–6.090	0.055
Undergraduate	0.762	2.142	0.905–5.072	0.083
Graduate/postgraduate	RC			
**Cadre**				
Community health worker	−0.276	0.759	0.452–1.274	0.297
Nurse	RC			
**Facility level**				
Primary	−2.333	0.097	0.041–0.232	**0.001**
Secondary	−0.902	0.406	0.176–0.936	0.034
Tertiary	RC			
**Senatorial district**				
Kano South	−0.689	0.502	0.234–1.076	
Kano North	−0.419	0.658	0.318–1.362	0.260
Kano Central	RC			

RC = Reference category.

### Assessment of the state of preparedness of the healthcare facilities

Assessment checklist for evaluating the state of preparedness for diphtheria using the variables: training and education, emergency preparedness and response, vaccination and immunization, surveillance and reporting, infection and control, and medication and treatment. The state of facility preparedness for diphtheria responses were poor, with 70.8% of the facilities, 20.8% being moderate, and 8.3% being good ( Table 6).

**Table 6.** Assessment checklist for evaluating the state of preparedness of facility for diphtheria response.

Parameters	Yes (%)	No (%)
**Emergency response plan**		
The facility established a comprehensive emergency response plan specific to diphtheria	13 (54.2)	11 (45.8)
The plan routinely reviewed and revised is as necessary	14 (56.3)	10 (41.7)
The roles and responsibilities of staff are clearly stated within the plan	15 (62.5)	9 (37.5)
There are designated individual responsible for overseeing the response efforts	16 (66.7)	8 (33.3)
**Training and education**		
All healthcare personnel have received training on the protocols associated with diphtheria response	7 (29.2)	17 (70.8)
Staff members are knowledgeable about the symptoms and signs of diphtheria	23 (95.8)	1 (4.2)
Staffs have been adequately trained in the proper utilization of personal protective equipment (PPE)	23 (95.8)	1 (4.2)
There is an ongoing program for staff training and refresher courses on diphtheria	10 (41.7)	14 (58.3)
**Vaccination and immunization**		
The vaccination coverage target is met in the facility for the pentavalent vaccine against diphtheria	11 (45.8)	13 (54.2)
There are systems in place for maintaining accurate records of the vaccination status of children	13 (54.2)	11 (45.8)
The facility has protocols in place for administering booster vaccinations as necessary for diphtheria	12 (50.0)	12 (50.0)
**Surveillance and reporting**		
The facility possesses a robust system for monitoring diphtheria cases within the community	14 (58.3)	10 (41.7)
There is a streamlined process for promptly reporting suspected cases to local health authorities	23 (95.8)	1 (4.2)
The facility is linked to a regional or national disease surveillance network	24 (100.0)	0 (0.0)
**Infection control**		
There is a designated isolation area for diphtheria cases	7 (29.2)	17 (70.8)
Hand hygiene stations readily available throughout the facility	15 (62.6)	9 (37.5)
The facility possesses an ample supply of personal protective equipment (PPE) for healthcare staff	22 (91.7)	2 (8.3)
Stringent hand hygiene and infection control measures is in place	22 (91.7)	2 (8.3)
There are written infection prevention and control measures for diphtheria cases	19 (79.2)	5 (8.3)
**Medication and treatment**		
There are readily accessible stockpile of diphtheria antitoxin and antibiotics within the facility	12 (50.0)	12 (50.0)
There is an established process for promptly administering antitoxin and antibiotics to confirmed cases	9 (37.5)	15 (62.5)
Healthcare personnel have been trained in the correct administration of these medications	8 (33.3)	16 (66.7)

## Discussion

Ensuring epidemic preparedness and response play an important role in every sovereign nation. Every threat to health, particularly the spread of communicable diseases, constitutes a potential threat to human existence. During infectious disease outbreaks, healthcare professionals play a critical role in lowering morbidity and mortality rates while also providing routine medical care for other patients. The public expects health workers to be able to maintain the health system’s functionality and offer surge capacity during infectious disease epidemics because of their moral and professional obligations to their patients ( Okoeguale et al., 2021). The success of any effort initiated to address epidemics within a country will primarily hinge on the proficiency, accessibility, and dedication of frontline healthcare workers.

The distribution of study respondents by sociodemographic variables revealed that the mean age of the respondents ranged from 34 ± 9.2. This is similar to a study on health workers in Northern Nigeria where the mean age of the respondents studied was 34.09 ± 9.1 ( Amoran and Onwube, 2013). The survey revealed that 54.7% of the participants were female and 45.8% were male. Many of the respondents identified as Muslims, accounting for 80.5%, while Christians made up 19.5%. The predominant ethnic group among the respondents was the Hausa, reflecting the ethnic distribution in Kano and the people’s predominant language. In terms of marital status, most respondents were married. In addition, 47.7% held a certificate or diploma, 8.3% had an undergraduate degree, and 43.9% had a graduate or postgraduate degree. Furthermore, 46.1% of the respondents were Community Health Extension Workers and slightly over half 53.9% were nurses.

Inadequate knowledge is a risk factor for disease transmission as it can lead to increased levels of care. The study demonstrated an overall knowledge score, showing that slightly above half of the respondents 51% had poor knowledge of diphtheria as compared to 49% of the respondents who had good knowledge. This is in contrast to a study carried out in Nepal and Ghana ( Amoran and Onwube, 2013; Upadhyaya et al., 2020) but similar to a study carried out in Libya, healthcare workers had poor knowledge ( Nkansah et al., 2020). This knowledge gap might be the result of the disease not being common, lack of interest in the disease over the years, and the fact that it is a vaccine-preventable disease.

The results regarding healthcare workers’ knowledge of diphtheria indicated that 97.6% acknowledged the disease exists. A significant majority understand that diphtheria can reoccur, and that they exhibit a strong grasp of transmission methods, correctly identifying sneezing, coughing, and contact with open sores. Preventive measures were well understood, with the majority emphasizing personal hygiene and recognizing the importance of vaccination. However, some misconceptions exist, as certain respondents associated diphtheria prevention with activities, such as regular bathing and insecticide use. Symptom awareness was high among healthcare workers, as they identified sore throat, difficulty breathing, and fever as key indicators. They were also knowledgeable about potential complications, such as suffocation and myocarditis. In terms of treatment, the majority (96.2%) correctly identified antibiotics as a suitable option and 86.2% acknowledged the role of antitoxins. The majority also acknowledged health workers’ susceptibility and routine vaccination for diphtheria at 6,10 and 14 weeks. Nevertheless, some misconceptions persist, such as a notable number of incorrectly associated topical creams, anti-hypertensives, and anti-rabies drugs with diphtheria treatment. Another primary concern emerging from this study was that only 35.7% knew that a single case could result in an outbreak, while majority believed cluster of cases and an incidence greater than expected was criteria for outbreaks. This reveals that there is a knowledge gap among health workers regarding the criteria that define an outbreak of diphtheria. In addition, only 25.4% of the respondents could provide the case definition of a suspected case of diphtheria recommended by WHO. These misconceptions therefore underscore the need for targeted education and clarification efforts regarding the disease among healthcare workers in general.

Healthcare workers are more vulnerable to contracting infectious diseases, especially in cases in which strict adherence to standard precautions is lacking. There is documented evidence that healthcare professionals acquire infections from patients under their care, resulting in some recorded fatalities ( Nkansah et al., 2020). They are more vulnerable to contracting infectious diseases, especially in cases in which strict adherence to standard precautions is lacking. However, in situations without epidemics, healthcare professionals fail to consistently adhere to the WHO’s recommended standard precautions, which could increase their vulnerability to contracting infectious diseases and spread them when managing suspected or confirmed cases. The level of infection prevention control practices among the respondents in this study was poor; slightly above half (53.4%) of the respondents had poor practice as compared with 46.6% of the respondents who had good practice. This is in contrast with a study finding in Rwanda, where 63.3% of respondents had good practice towards IPC, while 36.7 percent had poor practices. The overall mean practice score was 11.8 (SD = 2.1) ( Haguminshuti et al., 2022).

## Conclusions

Epidemic preparedness is important in responding to an outbreak and from this study it was revealed that the healthcare workers had poor knowledge of diphtheria as well as poor infection prevention control practices. These factors are crucial in disease outbreaks. The study identified key predictors, such as age groups between 26–40 years, educational levels including certificate/diploma and undergraduate education levels, as well as specific ethnic groups (Fulani, Hausa, Igbo, Yoruba), marital status, cadre, facility level, and senatorial districts. Additionally, political instability, logistic factors, and training and re-training were highlighted as important considerations.

### Recommendations

1. Funding needs to be generated and set aside in anticipation and preparation for any disease outbreak, rather than only to mitigate disease outbreaks.
2. Training and re-training of health workers on the knowledge of disease diphtheria and IPC practices should be constantly and consistently done, as they are always in contact with patients.
3. Provision of adequate PPEs should always be in constant supply in health facilities, and health workers should be encouraged to wear them.
4. Health Education should be made a continuous effort so that HCWs are always alert and not loose guards. This will help to avoid panic and the fire-brigade approach when disease outbreaks occur.

## Data Availability

Zenodo: Preparedness for diphtheria epidemic among healthcare workers in Kano State, Nigeria. Zenodo; 2025, https://doi.org/10.5281/zenodo.17887775 ( Ayodele, 2025). This project contains the following underlying data:
• Preparedness Diphtheria.sav Data are available under the terms Creative Commons Attribution 4.0 International (CC BY 4.0). Zenodo: Preparedness for diphtheria epidemic among healthcare workers in Kano State, Nigeria. Zenodo; 2025, https://doi.org/10.5281/zenodo.17887775 ( Ayodele, 2025). This project contains the following underlying data:
• Consent form and Questionnaire.docx • Ethical approval document.pdf Data are available under the terms Creative Commons Attribution 4.0 International (CC BY 4.0).

## Software availability statement

All software used to analyse data in this article are open access. The Statistical Package for Social Sciences (SPSS) version 20 and Stata version 12.1 were licensed to and provided by the Department of Epidemiology and Medical Statistics, University of Ibadan, Nigeria (Serial Number: 40120576630).

Data Citation: Ayodele F, *et al*. Preparedness for diphtheria epidemic among healthcare workers in Kano State, Nigeria. Zenodo; 2025, https://doi.org/10.5281/zenodo.17887775
