# Preparedness for diphtheria epidemic among healthcare workers in Kano State, Nigeria

**DOI:** 10.12688/openreseurope.22188.2

**Published:** 2026-07-21

**Authors:** Ayodele Arinola Feyisara, Noah David, Etim Utibe Efre, Kevin Dofwah Nuhu, Azibadighi Walter, Olufunmilayo Ibitola Fawole, Adeniyi Francis Fagbamigbe, Segun Bello, Onoja Matthew Akpa

**Affiliations:** 1 Epidemiology and Medical Statistics, University of Ibadan Faculty of Public Health, Ibadan, Oyo, Nigeria; 2 Epidemiology and Community Health, University of Ilorin Teaching Hospital, Ilorin, Kwara, Nigeria; 3 Fellow, European and Developing Countries Training Program (EDCTP) MPH EBDOER, Ibadan, Nigeria; 4 Pathology, Aminu Kano Teaching Hospital, Kano, Kano, Nigeria; 5 Veterinary and Pest Control Services, Ministry of Agriculture, Uyo, Akwa Ibom, Nigeria; 6 Department of Epidemiology and Medical statistics, College of Medicine, University of Ibadan, Ibadan, Oyo, Nigeria

**Keywords:** Diphtheria, healthcare workers, preparedness

## Abstract

Diphtheria remains a significant public health threat in Nigeria, highlighting the need for an adequately prepared healthcare workforce and health system. This study assessed healthcare workers’ knowledge of diphtheria, infection prevention and control (IPC) practices, and preparedness for diphtheria outbreaks in Kano State, Nigeria. A descriptive cross-sectional study was conducted among 421 healthcare workers selected using a multistage sampling technique. Data were collected using a semi-structured interviewer-administered questionnaire, while facility preparedness was assessed using a structured checklist completed by designated facility focal persons in 24 selected health facilities. Descriptive statistics and logistic regression analyses were performed at a 5% significance level using SPSS version 20. Results showed that Overall, 51.0% of healthcare workers had poor knowledge of diphtheria, while 53.4% demonstrated poor IPC practices. Healthcare workers in primary healthcare facilities were significantly less likely to have good IPC practices than those in tertiary facilities (OR = 0.097, 95% CI: 0.041–0.232; p < 0.001). Although respondents demonstrated good individual preparedness for diphtheria outbreaks, 70.8% of health facilities were classified as poorly prepared. Conclusions, despite good self-reported individual preparedness among healthcare workers, important deficiencies in knowledge, IPC practices, and facility preparedness remain. Strengthening healthcare worker training, IPC implementation, and facility-level outbreak preparedness is essential to improve readiness for future diphtheria outbreaks.

**List of abbreviations**
HCWs: Healthcare Workers
IPC: Infection Prevention Control
IHR: International Health Regulations
IDSR: Integrated Disease Surveillance and Response
SPSS: Statistical Package for the Social Sciences

## Background

Diseases transcend national borders, making global health security a shared responsibility. Recent outbreaks have highlighted the interconnected nature of health systems and demonstrated how infectious disease threats in, one region can rapidly affect populations worldwide ( CDC, 2023a). Emergence and re-emerging infectious diseases are among the leading threats to global health security, and one way to combat this is by workforce development of frontline staff to identify, track and contain outbreaks at their source. The impact of infectious disease outbreaks is amplified when investments in global health security are inadequate. The goal of the global health security agenda is to improve the global capacity to prevent, identify, and address infectious disease threats. Nigeria is among the countries that have signed this framework. In 2019, Nigeria became a global health security agenda partner country committed to improving progress towards global health security agenda goals and internal health regulations ( CDC, 2023b).

Preparedness is a requirement of 196 signatories to the International Health Regulations (IHR) (2005), the world’s defense against significant threats to public health. Systems must be capable of preventing, defending against, controlling, and responding to the international spread of diseases, which can occur at unanticipated rates in an interconnected world ( WHO, 2016). Epidemic preparedness refers to all the steps that are needed to be performed at the local, state, and federal levels, as well as in health facilities to be prepared and successfully address disease outbreaks. To prepare for an epidemic, routine surveillance systems must be developed to detect outbreaks, and employees must be organized to confirm, examine, and respond to epidemics ( Fatiregun and Isere, 2017). To deal with the disease, it is important to prepare a culture of safe medical procedures that can limit and stop the spread of disease outbreaks. Without a culture of safe practices, healthcare facilities will be more vulnerable to communicable disease exposure and health disruption ( Ughasoro et al., 2019).

In this study, preparedness for diphtheria outbreaks was conceptualized as the capacity of healthcare workers and health facilities to effectively prevent, detect, and respond to diphtheria outbreaks. Preparedness was assessed at two levels: individual preparedness among healthcare workers and facility preparedness. Knowledge of diphtheria and infection prevention and control (IPC) practices were considered determinants of preparedness rather than components of preparedness itself. Adequate knowledge facilitates timely outbreak recognition and response, while good IPC practices reduce transmission risks and strengthen response capacity. Recent WHO guidance emphasizes that suspected diphtheria cases should be identified and treated promptly without waiting for laboratory confirmation. Recommended management includes administration of diphtheria antitoxin, appropriate antibiotic therapy, isolation of cases, and implementation of infection prevention and control (IPC) measures, including droplet precautions, hand hygiene, environmental cleaning, and WASH interventions. These measures are essential for reducing transmission, improving patient outcomes, and protecting healthcare workers during outbreaks ( WHO, 2024a, 2024b).

**Figure 1.**
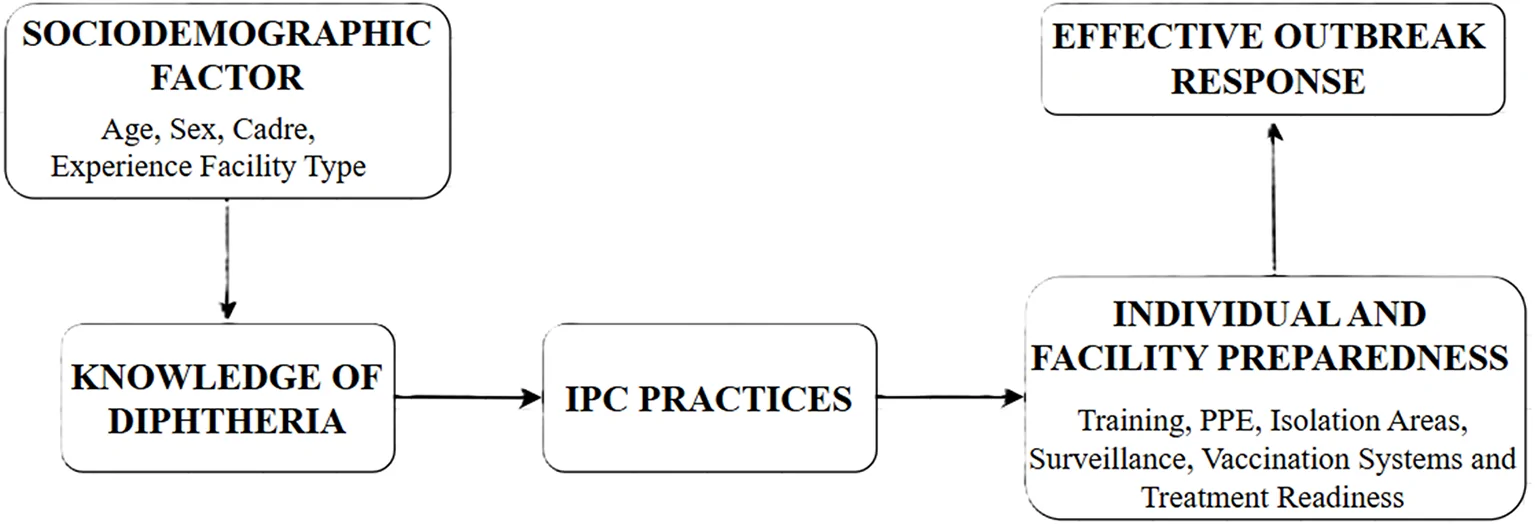
Conceptual framework showing the relationship between knowledge, IPC practices, individual preparedness, and facility preparedness for diphtheria outbreak response.

Diphtheria, a vaccine-preventable disease and an epidemic-prone notifiable disease reported under the integrated disease surveillance and response system (IDSR), has been a global burden that has improved with the introduction of vaccines into immunization programs. However, in Nigeria today, there is a surge in the incidence of this contagious disease, causing epidemics that primarily affect children. It is caused by Corynebacterium spp., mainly by toxin-producing *Corynebacterium diphtheriae* and rarely by toxin-producing strains of *Corynebacterium ulcerans* and *Corynebacterium pseudotuberculosis.* Diphtheria commonly presents as tonsillitis, laryngitis, or pharyngitis with signs and symptoms such as fever, cough, running nose, sore throat, and red eyes, and is characterized by an adherent pseudo-membrane, a thick, grey coating made up of necrotic tissue and bacteria, which is the tell-tale sign of infection covering the tonsils, pharynx, and/or nose. In severe cases, complications such as, myocarditis and neuritis may occur.

The resurgence of diphtheria outbreaks has been linked to sub-optimal vaccine coverage. Healthcare workers are particularly vulnerable during diphtheria outbreaks because of their close contact with suspected and confirmed cases. Current recommendations for healthcare personnel include maintaining up-to-date diphtheria vaccination status, adherence to droplet and contact precautions, use of personal protective equipment, post-exposure prophylaxis where indicated, microbiological screening, symptom monitoring, and temporary work restrictions for infected personnel. Consequently, healthcare worker knowledge and IPC practices are critical determinants of outbreak preparedness ( CDC, 2024).

As of epidemiological week 44 of 2023, a cumulative total of 10,863 confirmed diphtheria cases and 560 deaths had been reported across 130 local government areas in 20 Nigerian states, with a case fatality of 5.2%. The immunization coverage in northern Nigeria rate ranged from 5–48% in 2018, with Kano state among the affected states reporting low coverage levels ( Eze et al., 2021). Kano State located in northwestern Nigeria, is currently one of the states most affected by the ongoing diphtheria outbreak, and has recorded the highest number of cases and deaths.

Recent epidemiological analyses have further highlighted the magnitude of the Kano outbreak. An analysis of 18,320 confirmed diphtheria cases published in EBioMedicine documented substantial morbidity and mortality and identified persistent immunity gaps and delayed healthcare seeking as important contributors to transmission. Similarly, a study published on Infectious Disease Modelling study examined the transmission dynamics of diphtheria in Kano and demonstrated the importance of rapid case detection, vaccination, and outbreak control measures in reducing transmission ( Abbas et al., 2025; Kamadjeu et al., 2025). These findings emphasize the need for a well-prepared healthcare workforce capable of early case recognition, timely reporting, and effective case management. Skilled healthcare personnel are indispensable for successful infectious disease outbreak response. Therefore, this study aimed to assess healthcare workers’ knowledge of diphtheria, infection prevention and control practices, and preparedness for diphtheria outbreaks in Kano State, Nigeria.

## Methodology

### Study design and setting

A descriptive cross-sectional study was conducted between September and December 2023 among healthcare workers in Kano State, northwestern Nigeria, one of the states most affected by the recent resurgence of diphtheria. Kano State has an estimated population of approximately 13.1 million people and comprises 44 Local Government Areas (LGAs) distributed across three senatorial districts. Public health services are delivered through primary, secondary, and tertiary healthcare facilities and coordinated by the Kano State Ministry of Health.

Preparedness was conceptualized as the capacity of healthcare workers and health facilities to effectively prevent, detect, and respond to diphtheria outbreaks. Preparedness was assessed at both individual and facility levels, while knowledge of diphtheria and infection prevention and control (IPC) practices were evaluated as determinants of preparedness.

### Study population

The study population comprised Nurses and Community Health Extension Workers (CHEWs) employed in registered public primary, secondary, and tertiary healthcare facilities in Kano State. Eligible participants were healthcare workers present during data collection who provided written informed consent. Administrative staff, volunteers, interns, and healthcare workers absent during the survey period were excluded.

### Sample size

The sample size was calculated using the single population proportion formula for descriptive cross-sectional studies, assuming a prevalence of good IPC practices of 56%, a 95% confidence level, and a 5% margin of error. After adjustment for a 10% non-response rate, a final sample size of 421 healthcare workers was obtained.

The minimum sample size was calculated using the single population proportion formula for descriptive cross-sectional studies:

$$n=\frac{\;Z^{2}\mathrm{pq}}{d^{2}}$$

where:

• *Z* = 1.96 corresponding to a 95% confidence level;
• *p* = The sample size was calculated using a prevalence of 56% ( *p* = 0.56), representing the proportion of healthcare workers with good infection prevention and control practices reported in a previous study among healthcare workers in Nigeria ( Amali & Van Wyk, 2023).
• *q* = 1 – *p* = 0.44;
• *d* = 0.05 margin of error.

The calculated sample size was 379 participants. After adjusting for a 10% non-response rate, the final sample size was increased to 421 healthcare workers.

### Sampling

A multistage sampling technique was used. Kano State was stratified into its three senatorial districts, from which one LGA was selected per district. One LGA in Kano Central containing a tertiary healthcare facility was purposively selected, while the remaining LGAs were selected by simple random sampling. Subsequently, health facilities were selected through simple random sampling, except for the tertiary facility, which was included purposively. Proportionate allocation was used to distribute the sample across senatorial districts, and eligible healthcare workers were recruited consecutively within selected facilities until the required sample size was achieved. Facility preparedness was assessed in 24 health facilities selected through simple random sampling from eligible facilities within the selected LGAs.

### Data collection

Data were collected using a semi-structured interviewer-administered questionnaire adapted from the World Health Organization (WHO) Diphtheria Surveillance Standards, WHO Diphtheria Fact Sheet, Nigeria Integrated Disease Surveillance and Response (IDSR) Guidelines, and the WHO Infection Prevention and Control Assessment Framework. The questionnaire assessed socio-demographic characteristics, knowledge of diphtheria, IPC practices, and preparedness for diphtheria outbreaks. Eight trained research assistants collected data under the supervision of the principal investigator. The questionnaire was developed in English and administered through face-to-face interviews. Research assistants were fluent in both English and Hausa and conducted interviews in the participant’s preferred language. Where necessary, questions were verbally explained in Hausa to facilitate understanding. A structured facility preparedness checklist, adapted from WHO and NCDC guidelines, was completed by the designated focal person in each facility. Information was obtained through self-report and verified, where possible, by review of available records and facility resources. The checklist assessed the availability of preparedness guidelines, surveillance tools, personal protective equipment, laboratory capacity, isolation facilities, essential supplies, and staff training.

### Measurement of variables

**Scoring and classification:** Responses were scored and aggregated to generate composite scores for knowledge, IPC practices, facility preparedness. Classification thresholds were based on percentile cut-offs and previously published assessment approaches ( Haguminshuti et al., 2022; WHO, 2015).

**Knowledge of Diphtheria:** Knowledge was assessed using 45 items covering disease transmission, symptoms, prevention, surveillance, outbreak recognition, and treatment. The median score (28/45) was used as the cut-off point for classification, consistent with previous studies ( Haguminshuti et al., 2022). Respondents scoring 0–28 were classified as having poor knowledge, while those scoring 29–45 were classified as having good knowledge.

**Infection Prevention and Control Practices:** IPC practices were assessed using 19 items. The median score (14/19) was used as the cut-off point for classification ( Haguminshuti et al., 2022). Respondents scoring 0–14 were classified as having poor IPC practices, while those scoring 15–19 were classified as having good IPC practices.

**Facility Preparedness:** Facility preparedness was assessed using a 33-item checklist covering emergency preparedness and response, staff training, vaccination services, surveillance systems, infection prevention and control measures, and treatment readiness. Preparedness was categorized as poor (0–11), moderate (12–22), or good (23–33), based on percentile thresholds adapted from the WHO Service Availability and Readiness Assessment framework ( WHO, 2015).

### Instrument validation and pretesting

The study instruments were reviewed by experts in epidemiology, public health, and disease surveillance to establish content validity. Pretesting was conducted among healthcare workers in Bebeji LGA, which was not included in the main study, using approximately 10% of the calculated sample size. Feedback from the pretest informed revisions to improve clarity, wording, and administration procedures. The internal consistency of the knowledge assessment items was evaluated using Cronbach’s alpha coefficient. The knowledge and infection prevention scale yielded a Cronbach’s alpha of 0.8410 and 0.7701 respectively, indicating acceptable reliability.

### Statistical analysis

Data were analysed using IBM SPSS Statistics version 20. Descriptive statistics were used to summarize respondents’ socio-demographic characteristics and study variables using frequencies, percentages, means, and standard deviations, as appropriate. Associations between independent variables and knowledge of diphtheria and infection prevention and control (IPC) practices were assessed using Pearson’s chi-square test. Variables with p-values <0.20 in the bivariate analysis and those considered epidemiologically relevant based on previous literature were entered into separate multivariable binary logistic regression models. Knowledge and IPC practice outcomes were coded as binary variables (good = 1, poor = 0). Adjusted odds ratios (AORs) with 95% confidence intervals (CIs) were reported, and statistical significance was set at p < 0.05. Facility preparedness was summarised descriptively using frequencies and percentages. Categories with sparse observations were combined where necessary to improve model stability.

### Ethical approval statement and consent to participate

Ethical approval was obtained from the Health Research Ethics Committee of the Kano State Ministry of Health, Kano State, Nigeria on the 19 ^th^ of May 2023.The approval number is NHREC/17/03/2018 and the reference number is SHREC/2023/3985.The study commenced in September 2023 where written informed consent was obtained from all the participants. No harm was caused to the participants during the study Participants were informed about the choice to participate or withdraw from the study at any time with no consequences.

### Consent to participate

A written informed consent was obtained from all participants, and this document was approved by the ethics committee before the study was implemented.

## Results

The mean age of the healthcare workers was 34 ± 9.2 years, indicating that most participants were in their early to mid-adulthood. Females constituted slightly more than half of the respondents (54.2%), while males accounted for 45.8%. The majority of participants were Muslims (80.5%) and of Hausa ethnicity (58.9%), reflecting the predominant religious and ethnic composition of Kano State. Regarding educational attainment, 47.7% of respondents held a certificate or diploma, 8.3% had an undergraduate degree, and 44.0% had a graduate or postgraduate qualification. Most respondents were married (72.5%), while 26.1% had never married. Nurses constituted 53.9% of the study population, whereas 46.1% were Community Health Extension Workers (CHEWs). Respondents were fairly distributed across the three senatorial districts, with 31.4%, 33.0%, and 35.6% drawn from Kano South, Kano North, and Kano Central, respectively. In terms of facility level, 47.0% worked in primary healthcare facilities, 25.9% in secondary healthcare facilities, and 27.1% in tertiary healthcare facilities (  Table 1).

**Table 1.** Socio-demographic characteristics of the respondents.

Socio-demographics	Frequency
**Age group (years)**	
≤ 25	83 (19.7)
26–30	113 (26.8)
31–35	77 (18.3)
36–40	62 (14.7)
41+	86 (20.5)
Mean age ± SD (years)	34 ± 9.2
**Sex**	
Female	228 (54.2)
Male	193 (45.8)
**Highest level of education**	
Certificate/diploma	201 (47.7)
Undergraduate	35 (8.3)
Graduate/postgraduate	185 (44.0)
**Religion**	
Islam	339 (80.5)
Christianity	82 (19.5)
**Ethnicity**	
Fulani	75 (17.8)
Hausa	248 (58.9)
Igbo	55 (13.1)
Yoruba	35 (8.3)
Others	8 (1.9)
**Marital status**	
Never married	110 (26.1)
Married	305 (72.5)
Divorced/separated/widowed	6 (1.4)
**Cadre**	
Community health worker	194 (46.1)
Nurse	227 (53.9)
**Facility level**	
Primary	198 (47.0)
Secondary	109 (25.9)
Tertiary	114 (27.1)
**Senatorial district**	
Kano South	132 (31.4)
Kano North	139 (33.0)
Kano Central	150 (35.6)

### Overall knowledge grading of respondents

For the overall knowledge score, slightly above half of the respondents 51% have poor knowledge as compared with 49% of the respondents who had good knowledge ( Figure 2).

**Figure 2.**
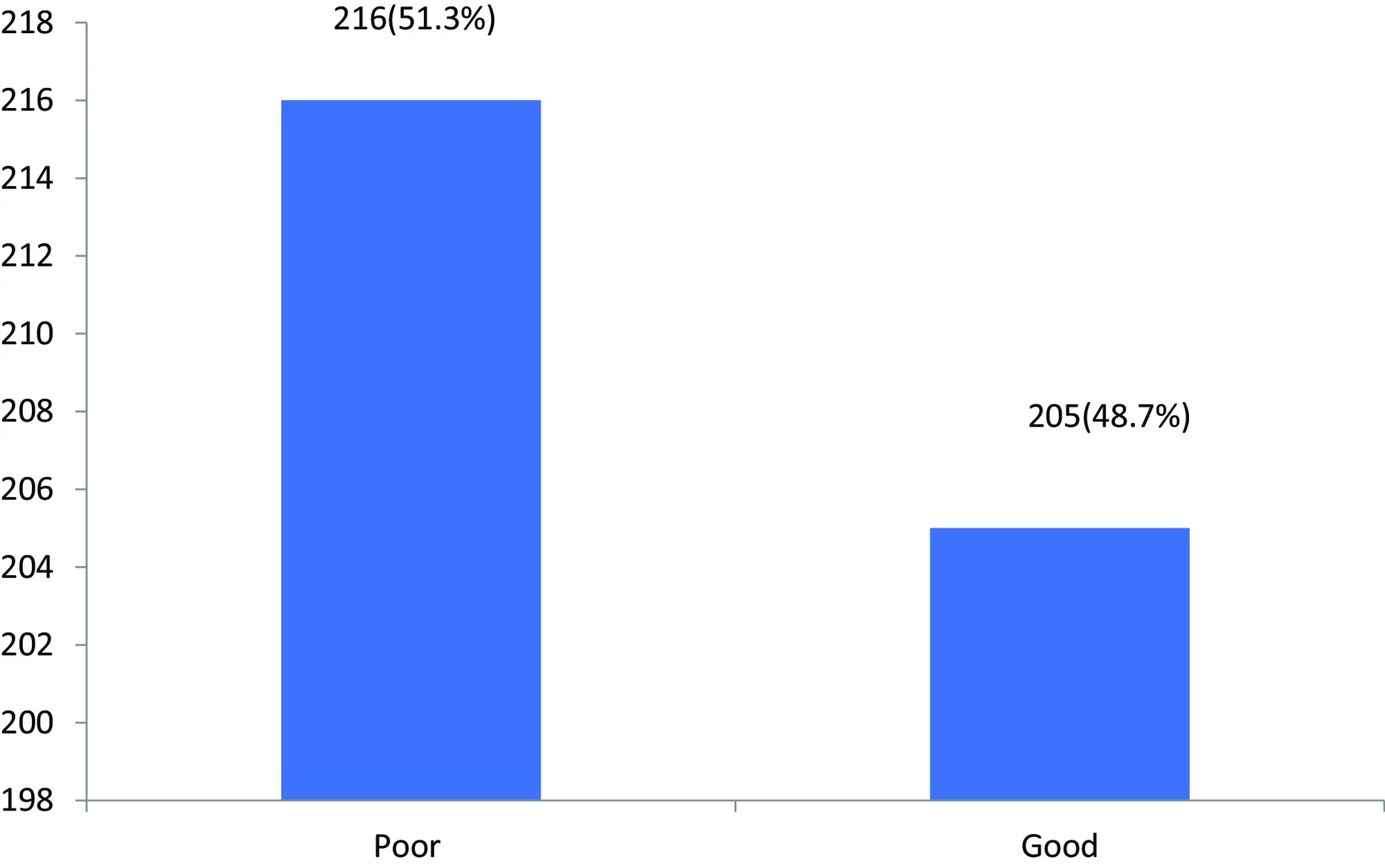
The level of Knowledge among respondents.

### Respondents’ association between socio-demographic characteristics and knowledge of diphtheria

The distribution of knowledge of diphtheria according to respondents’ background characteristics is presented in Table 2. Knowledge was significantly associated with age group (χ ^2^ = 15.323, p = 0.004), highest educational level (χ ^2^ = 8.001, p = 0.018), religion (χ ^2^ = 7.431, p = 0.006), ethnicity (χ ^2^ = 11.907, p = 0.018), and marital status (χ ^2^ = 6.779, p = 0.028). Higher proportions of good knowledge were observed among respondents with graduate/postgraduate education (56.2%), Christians (62.2%), and respondents of other ethnicities (75.0%). No significant associations were found between knowledge and sex (p = 0.560), cadre (p = 0.144), facility level (p = 0.118), or senatorial district (p = 0.247) ( Table 2).

**Table 2.** Association between Socio-demographic characteristics and knowledge of diphtheria among Healthcare Workers.

Parameters	Knowledge			
	Poor (%)	Good (%)	χ ^2^	p-value
**Age group (years)**			15.323	0.004*
≤ 25	57 (68.7)	26 (31.3)		
26–30	50 (44.2)	63 (55.8)		
31–35	42 (54.5)	35 (45.5)		
36–40	26 (41.9)	36 (58.1)		
> 40	41 (47.7)	45 (52.3)		
**Sex**			0.340	0.560
Female	114 (50.0)	114 (50.0)		
Male	102 (52.8)	91 (47.2)		
**Highest level of education**			8.001	0.018*
Certificate/diploma	113 (56.2)	88 (43.8)		
Undergraduate	22 (62.9)	13 (37.1)		
Graduate/postgraduate	81 (43.8)	104 (56.2)		
**Religion**			7.431	0.006*
Islam	185 (54.6)	154 (45.4)		
Christianity	31 (37.8)	51 (62.2)		
**Ethnicity**			11.907	0.018*
Fulani	38 (50.7)	37 (49.3)		
Hausa	141 (56.9)	107 (43.1)		
Igbo	19 (34.5)	36 (65.5)		
Yoruba	16 (45.7)	19 (54.3)		
Others	2 (25.0)	6 (75.0)		
**Marital status**			6.779	**0.028 ^f^**
Not married	66 (56.9)	50 (43.1)		
Married	150 (49.2)	155 (50.8)		
**Cadre**			2.133	0.144
Community health worker	107 (55.2)	87 (44.8)		
Nurse	109 (48.0)	118 (52.0)		
**Facility level**			4.278	0.118
Primary	91 (46.0)	107 (54.0)		
Secondary	61 (56.0)	48 (44.0)		
Tertiary	64 (56.1)	50 (43.9)		
**Senatorial district**			2.796	0.247
Kano South	62 (47.0)	70 (53.0)		
Kano North	79 (56.8)	60 (43.2)		
Kano Central	75 (50.0)	75 (50.0)		

### Respondents’ association between socio-demographic characteristics and infection prevention control practices

The distribution of IPC practices according to respondents’ background characteristics is presented in  Table 3. Good IPC practice was significantly associated with highest educational level (χ ^2^ = 12.395, p = 0.002), cadre (χ ^2^ = 15.862, p = 0.001), healthcare facility level (χ ^2^ = 108.454, p = 0.001), and senatorial district (χ ^2^ = 66.408, p = 0.001). Higher proportions of good IPC practice were observed among respondents with graduate/postgraduate education (56.2%), nurses (55.5%), those working in tertiary healthcare facilities (81.6%), and respondents from Kano Central Senatorial District (72.0%). No significant associations were found between IPC practice and age group (p = 0.218), sex (p = 0.341), ethnicity (p = 0.404), or marital status (p = 0.425) (  Table 3).

**Table 3.** Association between socio-demographic characteristics and infection prevention control practice of diphtheria among healthcare workers.

Variables	Practice		χ ^2^	p-value
	Poor (%)	Good (%)		
**Age group (years)**			5.762	0.218
≤ 25	47 (56.6)	36 (43.4)		
26–30	67 (59.3)	46 (40.7)		
31–35	40 (51.9)	37 (48.1)		
36–40	34 (54.8)	28 (45.2)		
> 40	37 (43.0)	49 (57.0)		
**Sex**			0.905	0.341
Female	117 (51.3)	111 (48.7)		
Male	108 (56.0)	85 (44.0)		
**Highest level of education**			12.395	0.002*
Certificate/diploma	123 (61.2)	79 (38.8)		
Undergraduate	21 (60.0)	14 (40.0)		
Graduate/postgraduate	81 (43.8)	104 (56.2)		
**Ethnicity**			4.018	0.404
Fulani	41 (54.7)	34 (45.3)		
Hausa	134 (54.0)	114 (46.0)		
Igbo	32 (58.2)	23 (41.8)		
Yoruba	16 (45.7)	19 (54.3)		
Others	2 (25.0)	6 (75.0)		
**Marital status**			1.829	0.425 ^f^
Never married	55 (50.0)	55 (50.0)		
Married	168 (55.1)	137 (44.9)		
Divorced/separated/widowed	2 (33.3)	4 (66.7)		
**Cadre**			15.862	**0.001**
Community health worker	124 (63.9)	70 (36.1)		
Nurse	101 (44.5)	126 (55.5)		
**Facility level**			108.454	**0.001**
Primary	155 (78.3)	43 (21.7)		
Secondary	49 (45.0)	60 (55.0)		
Tertiary	21 (18.4)	93 (81.6)		
**Senatorial district**			66.408	**0.001**
Kano South	99 (75.0)	33 (25.0)		
Kano North	84 (60.4)	55 (39.6)		
Kano Central	42 (28.0)	108 (72.0)		

### Respondents’ socio-demographic predictors of knowledge of diphtheria

In the multivariable logistic regression analysis, only religion remained independently associated with knowledge of diphtheria. Muslim healthcare workers were less likely to have good knowledge than Christians (AOR = 0.43, 95% CI: 0.32–0.87; p = 0.012), while age, educational level, ethnicity, and marital status were not significant predictors (  Table 4).

**Table 4.** Socio-demographic predictors of knowledge of diphtheria among study participants.

Variables	β	AOR	95% CI	p-value
**Age group (years)**				
≤ 25	−0.561	0.570	0.280–1.161	0.121
26–30	0.334	1.397	0.757–2.579	0.285
31–35	−0.143	0.867	0.457–1.645	0.662
36–40	0.278	1.320	0.669–2.606	0.424
> 40	RC			
**Highest level of education**				
Certificate/diploma	0.445	1.561	0.715–3.408	0.264
Undergraduate	0.673	1.960	0.887–4.332	0.096
Graduate/postgraduate	RC			
**Religion**				
Islam	−0.638	0.428	0.322–0.868	**0.012**
Christianity	RC			
**Ethnicity**				
Fulani	−0.052	0.949	0.408–2.206	0.903
Hausa	−0.280	0.756	0.357–1.602	0.465
Igbo	0.493	1.637	0.668–4.010	0.281
Others	1.030	2.802	0.474–16.548	0.256
Yoruba	RC			
**Marital status**				
Never married	0.055	1.057	0.650–1.719	0.823
Married	RC			

RC = Reference category.

### Respondents’ predictors of infection prevention control practices

Table 5 presents the multivariable logistic regression analysis of factors associated with good IPC practice. After adjusting for potential confounders, only healthcare facility level remained independently associated with IPC practice. Compared with respondents working in tertiary healthcare facilities, those in primary healthcare facilities were significantly less likely to have good IPC practice (AOR = 0.097, 95% CI: 0.041–0.232, p = 0.001), while those in secondary healthcare facilities were also less likely to have good IPC practice (AOR = 0.406, 95% CI: 0.176–0.936, p = 0.034). Educational level, cadre, and senatorial district were not independently associated with IPC practice (p > 0.05) (  Table 5).

**Table 5.** Predictors of Infection prevention control practice of diphtheria among study respondents.

Variables	β	AOR	95% CI	p-value
**Highest level of education**				
Certificate/diploma	0.894	2.445	0.982–6.090	0.055
Undergraduate	0.762	2.142	0.905–5.072	0.083
Graduate/postgraduate	RC			
**Cadre**				
Community health worker	−0.276	0.759	0.452–1.274	0.297
Nurse	RC			
**Facility level**				
Primary	−2.333	0.097	0.041–0.232	**0.001**
Secondary	−0.902	0.406	0.176–0.936	0.034
Tertiary	RC			
**Senatorial district**				
Kano South	−0.689	0.502	0.234–1.076	
Kano North	−0.419	0.658	0.318–1.362	0.260
Kano Central	RC			

RC = Reference category.

### Assessment of the state of preparedness of the healthcare facilities

Facility preparedness for diphtheria was assessed across six domains: emergency preparedness and response, training and education, vaccination and immunization, surveillance and reporting, infection prevention and control, and medication and treatment readiness. Overall, preparedness was poor, with 70.8% of facilities classified as poorly prepared, 20.8% as moderately prepared, and only 8.3% as well prepared. While most facilities reported strong surveillance and reporting systems and adequate availability of personal protective equipment, important gaps were identified in staff training, availability of isolation areas, vaccination systems, and treatment readiness ( Table 6).

**Table 6.** Assessment checklist for evaluating the state of preparedness of facility for diphtheria response.

Parameters	Yes (%)	No (%)
**Emergency response plan**		
The facility established a comprehensive emergency response plan specific to diphtheria	13 (54.2)	11 (45.8)
The plan routinely reviewed and revised is as necessary	14 (56.3)	10 (41.7)
The roles and responsibilities of staff are clearly stated within the plan	15 (62.5)	9 (37.5)
There are designated individual responsible for overseeing the response efforts	16 (66.7)	8 (33.3)
**Training and education**		
All healthcare personnel have received training on the protocols associated with diphtheria response	7 (29.2)	17 (70.8)
Staff members are knowledgeable about the symptoms and signs of diphtheria	23 (95.8)	1 (4.2)
Staffs have been adequately trained in the proper utilization of personal protective equipment (PPE)	23 (95.8)	1 (4.2)
There is an ongoing program for staff training and refresher courses on diphtheria	10 (41.7)	14 (58.3)
**Vaccination and immunization**		
The vaccination coverage target is met in the facility for the pentavalent vaccine against diphtheria	11 (45.8)	13 (54.2)
There are systems in place for maintaining accurate records of the vaccination status of children	13 (54.2)	11 (45.8)
The facility has protocols in place for administering booster vaccinations as necessary for diphtheria	12 (50.0)	12 (50.0)
**Surveillance and reporting**		
The facility possesses a robust system for monitoring diphtheria cases within the community	14 (58.3)	10 (41.7)
There is a streamlined process for promptly reporting suspected cases to local health authorities	23 (95.8)	1 (4.2)
The facility is linked to a regional or national disease surveillance network	24 (100.0)	0 (0.0)
**Infection control**		
There is a designated isolation area for diphtheria cases	7 (29.2)	17 (70.8)
Hand hygiene stations readily available throughout the facility	15 (62.6)	9 (37.4)
The facility possesses an ample supply of personal protective equipment (PPE) for healthcare staff	22 (91.7)	2 (8.3)
Stringent hand hygiene and infection control measures is in place	22 (91.7)	2 (8.3)
There are written infection prevention and control measures for diphtheria cases	19 (79.2)	5 (8.2)
**Medication and treatment**		
There are readily accessible stockpile of diphtheria antitoxin and antibiotics within the facility	12 (50.0)	12 (50.0)
There is an established process for promptly administering antitoxin and antibiotics to confirmed cases	9 (37.5)	15 (62.5)
Healthcare personnel have been trained in the correct administration of these medications	8 (33.3)	16 (66.7)

## Discussion

This study assessed healthcare workers’ knowledge of diphtheria, infection prevention and control (IPC) practices, and preparedness for diphtheria outbreaks in Kano State, Nigeria. The findings revealed important gaps in knowledge and IPC practices despite relatively high levels of self-reported preparedness among healthcare workers. In addition, facility preparedness was generally poor, particularly in areas related to staff training, isolation capacity, vaccination systems, and treatment readiness. These findings are important in the context of the recent diphtheria resurgence in Kano State and suggest that significant weaknesses remain in the health system’s capacity to effectively prevent, detect, and respond to future outbreaks ( Abbas et al., 2025; Kamadjeu et al., 2025).

The study found that slightly more than half of the respondents had poor knowledge of diphtheria. Although many healthcare workers correctly identified common symptoms, modes of transmission, and the importance of vaccination, important deficiencies were observed in areas directly relevant to outbreak response. In particular, relatively few respondents correctly identified the WHO case definition of a suspected diphtheria case or recognized that a single confirmed case constitutes an outbreak requiring immediate public health action ( WHO, 2024a). These findings raise concerns because healthcare workers are often the first point of contact for patients and play a critical role in disease recognition, notification, case management, and implementation of control measures ( WHO, 2024a; CDC, 2024).

The observed knowledge gaps may partly reflect the epidemiology of diphtheria in Nigeria. For many years, the disease received limited attention compared with other priority infectious diseases, resulting in fewer training opportunities and lower familiarity among healthcare workers. However, the recent resurgence of diphtheria in Kano and other northern states has demonstrated the need for renewed attention to disease-specific training. Recent analyses of the Kano outbreak highlighted the contribution of delayed case recognition, immunity gaps, and late healthcare seeking to ongoing transmission ( Abbas et al., 2025). In such settings, inadequate knowledge among frontline healthcare workers may delay diagnosis and reporting, thereby reducing opportunities for early outbreak containment. Similar findings have been reported in studies examining preparedness for infectious disease outbreaks among healthcare workers in resource-constrained settings ( Okoeguale et al., 2021; Ughasoro et al., 2019).

For healthcare workers, disparities in knowledge may reflect unequal access to in-service training, differences in professional cadre, years of experience, and opportunities for participation in outbreak preparedness activities. Previous studies have shown that healthcare worker preparedness is influenced by prior training, disaster response experience, and access to capacity-building programmes ( Nayahangan et al., 2021; Balut et al., 2022). Targeted continuing professional development programmes, periodic simulation exercises, supportive supervision, and dissemination of updated clinical and public health guidelines may help address these gaps and strengthen preparedness for infectious disease outbreaks. Furthermore, simulation-based training has been demonstrated to improve healthcare workers’ knowledge, skills, confidence, and preparedness in outbreak response settings ( Kabi et al., 2021; Elendu et al., 2024). Ensuring equitable access to training across facility types and healthcare worker categories is particularly important in resource-constrained settings where preparedness depends heavily on frontline health personnel.

Regular in-service training, dissemination of updated clinical guidelines, and simulation exercises may therefore help address these gaps and improve outbreak response capacity ( WHO, 2024a).

Another important finding was the generally poor level of IPC practices among healthcare workers. Effective IPC remains one of the most important strategies for preventing healthcare-associated transmission during outbreaks. Current WHO recommendations emphasize rapid isolation of suspected cases, adherence to droplet precautions, appropriate use of personal protective equipment, hand hygiene, environmental cleaning, and other IPC measures as key components of diphtheria control ( WHO, 2024a, 2024b). Poor compliance with these practices may increase the risk of transmission within healthcare facilities and expose healthcare workers to preventable occupational infections.

Several factors may explain the poor IPC practices observed in this study. Although these factors were not directly assessed, challenges such as inadequate training, irregular supportive supervision, heavy workloads, and limited access to IPC resources have been reported in similar healthcare settings ( Haguminshuti et al., 2022; Ughasoro et al., 2019). The finding is particularly relevant because healthcare workers are at increased risk of exposure during outbreaks. CDC guidance for healthcare personnel emphasizes vaccination, post-exposure management, appropriate use of personal protective equipment, and strict adherence to infection prevention measures to reduce occupational transmission ( CDC, 2024). Strengthening IPC programmes through regular training, supervision, audits, and sustained availability of IPC supplies may therefore improve preparedness for future outbreaks ( WHO, 2024b; CDC, 2024).

A notable finding of this study was the substantially poorer IPC performance among healthcare workers in primary healthcare facilities compared with those in tertiary facilities. Respondents working in primary healthcare facilities were significantly less likely to demonstrate good IPC practices. This disparity is likely to reflect differences in infrastructure, access to continuing professional education, availability of IPC materials, staffing levels, and supervisory support across levels of care. Similar inequalities between primary and higher-level facilities have been reported in studies assessing outbreak preparedness and infection prevention practices in resource-constrained settings ( Fatiregun & Isere, 2017; Ughasoro et al., 2019).

The vulnerability of primary healthcare facilities has important implications for outbreak control. Primary healthcare facilities are often the first point of contact for patients with febrile illnesses and respiratory symptoms. Consequently, deficiencies in IPC practices at this level may contribute to delayed recognition of cases, inadequate infection control, and continued community transmission before referral occurs. Strengthening primary healthcare services should therefore be a priority for outbreak preparedness efforts. This includes ensuring adequate supplies of personal protective equipment, improving access to training, strengthening supervision, and integrating outbreak preparedness activities into routine service delivery ( Mearns et al., 2025).

The assessment of facility preparedness revealed that most facilities were inadequately prepared to respond to diphtheria outbreaks. The most prominent deficiencies were related to staff training, availability of designated isolation areas, vaccination monitoring systems, and treatment readiness. Although many facilities reported having surveillance and reporting structures in place, substantial weaknesses remained in other critical components of preparedness.

The limited availability of designated isolation areas is particularly concerning because prompt isolation of suspected cases is a cornerstone of diphtheria outbreak control ( WHO, 2024a, 2024b). Similarly, the absence of regular staff training programmes may reduce healthcare workers’ ability to identify cases early and implement appropriate response measures. The findings relating to treatment readiness are equally important. A considerable proportion of facilities lacked adequate arrangements for the prompt administration of diphtheria antitoxin and antibiotics, despite WHO recommendations that treatment should be initiated as early as possible because delays are associated with increased morbidity and mortality ( WHO, 2024a). Similar concerns regarding health facility readiness and outbreak response capacity have been reported in previous studies conducted in Nigeria ( Ughasoro et al., 2019).

Weaknesses in vaccination monitoring systems observed in this study may also have implications for outbreak prevention. The recent Kano outbreak demonstrated the consequences of immunity gaps within affected populations ( Abbas et al., 2025; Kamadjeu et al., 2025). Healthcare facilities play an important role in maintaining vaccination records, identifying missed children, and supporting routine immunization activities. Strengthening these systems may contribute not only to improved outbreak response but also to long-term prevention of future outbreaks ( Eze et al., 2021; Abbas et al., 2025).

Taken together, these findings indicate that preparedness for diphtheria outbreaks extends beyond individual healthcare worker knowledge. Effective preparedness requires a combination of competent healthcare workers, strong IPC systems, adequate treatment capacity, functional surveillance mechanisms, reliable vaccination services, and supportive institutional structures ( WHO, 2016, 2024a, 2024b; CDC, 2024). Weaknesses in any of these areas may compromise outbreak response efforts. Addressing the deficiencies identified in this study will require coordinated investments in workforce development, primary healthcare strengthening, IPC programmes, surveillance systems, and facility readiness.

### Strengths and limitations

A major strength of this study is that it assessed preparedness at both healthcare worker and facility levels across multiple tiers of the health system in one of the states most affected by the recent diphtheria outbreak in Nigeria. This provides a more comprehensive understanding of preparedness than studies focusing solely on individual healthcare workers. However, several limitations should be considered when interpreting the findings. The cross-sectional design does not permit causal inference. Knowledge, IPC practices, and individual preparedness were assessed using self-reported measures and may therefore be subject to recall and social desirability bias. In addition, the study included only nurses and community health extension workers in public health facilities and may not fully represent other categories of healthcare workers or private-sector facilities in Kano State.

### Recommendations

1. Institutionalize simulation exercises and outbreak preparedness drills at all levels of healthcare delivery to improve healthcare workers’ practical skills, confidence, and readiness to respond to diphtheria outbreaks.
2. Strengthen healthcare worker training on diphtheria preparedness and response through regular in-service training, continuing professional development programmes, and dissemination of updated national and WHO guidelines, with emphasis on case recognition, case definition, reporting procedures, and case management.
3. Enhance infection prevention and control (IPC) programmes, particularly in primary healthcare facilities, through regular supportive supervision, IPC audits, mentorship, and sustained availability of personal protective equipment and other IPC supplies.
4. Strengthen primary healthcare facilities, which demonstrated poorer IPC practices, by improving access to training opportunities, supervisory support, staffing, and essential outbreak response resources.
5. Conduct periodic preparedness assessments at both healthcare worker and facility levels to monitor progress, identify gaps, and guide targeted interventions for outbreak preparedness and response.
6. Improve facility preparedness for diphtheria outbreaks by establishing designated isolation areas, ensuring availability of diphtheria treatment supplies, strengthening vaccination monitoring systems, and maintaining functional outbreak response plans.

## Data Availability

Zenodo: Preparedness for diphtheria epidemic among healthcare workers in Kano State, Nigeria. Zenodo; 2025, https://doi.org/10.5281/zenodo.17887775 ( Ayodele, 2025). This project contains the following underlying data: • Preparedness Diphtheria.sav Data are available under the terms Creative Commons Attribution 4.0 International (CC BY 4.0). Zenodo: Preparedness for diphtheria epidemic among healthcare workers in Kano State, Nigeria. Zenodo; 2025, https://doi.org/10.5281/zenodo.17887775 ( Ayodele, 2025). This project contains the following underlying data: • Consent form and Questionnaire.docx • Ethical approval document.pdf Data are available under the terms Creative Commons Attribution 4.0 International (CC BY 4.0).

## Software availability statement

All software used to analyse data in this article are open access. The Statistical Package for Social Sciences (SPSS) version 20iiii were licensed to and provided by the Department of Epidemiology and Medical Statistics, University of Ibadan, Nigeria (Serial Number: 40120576630).
