# Supplementary material for: Preparedness for diphtheria epidemic among healthcare workers in Kano State, Nigeria
Source: Open Res Eur. 2026 Sep 15;6:107. Originally published 2026 Apr 13. [Version 3] doi: 10.12688/openreseurope.22188.3 (PMC13560850; doi:10.12688/openreseurope.22188.3)
Supplement: Peer review history

# Reviewer report: Reviewer response for version 3

**DOI:** 10.21956/openreseurope.27153.r81101

**Published:** 2026-09-17

**Authors:** Ukamaka Gladys Okafor

**Affiliations:** 1 Euclid University, Bangui, Central African Republic

The authors have comprehensively responded to the previous concerns I raised. The methods are clear and can be replicated.

Is the study design appropriate and does the work have academic merit?

Yes

Is the work clearly and accurately presented and does it cite the current literature?

Yes

If applicable, is the statistical analysis and its interpretation appropriate?

Yes

Are all the source data underlying the results available to ensure full reproducibility?

Yes

Are the conclusions drawn adequately supported by the results?

Yes

Are sufficient details of methods and analysis provided to allow replication by others?

No

Reviewer Expertise:

Public Health, Policy and regulation,

I confirm that I have read this submission and believe that I have an appropriate level of expertise to confirm that it is of an acceptable scientific standard.

# Reviewer report: Reviewer response for version 2

**DOI:** 10.21956/openreseurope.26461.r77755

**Published:** 2026-08-13

**Authors:** Ukamaka Gladys Okafor

**Affiliations:** 1 Euclid University, Bangui, Central African Republic

Dear Authors

 Your extensive revisions to the manuscript are commendable, and I am pleased with its current state.

Is the study design appropriate and does the work have academic merit?

Yes

Is the work clearly and accurately presented and does it cite the current literature?

Yes

If applicable, is the statistical analysis and its interpretation appropriate?

Yes

Are all the source data underlying the results available to ensure full reproducibility?

Yes

Are the conclusions drawn adequately supported by the results?

Yes

Are sufficient details of methods and analysis provided to allow replication by others?

No

Reviewer Expertise:

Public Health, Policy and regulation,

I confirm that I have read this submission and believe that I have an appropriate level of expertise to confirm that it is of an acceptable scientific standard.

# Reviewer report: Reviewer response for version 2

**DOI:** 10.21956/openreseurope.26461.r77753

**Published:** 2026-08-13

**Authors:** David Broderick

**Affiliations:** 1 The University of Auckland, Auckland, New Zealand

I thank the authors for addressing my concerns, the article should now be indexed.

Is the study design appropriate and does the work have academic merit?

Yes

Is the work clearly and accurately presented and does it cite the current literature?

Yes

If applicable, is the statistical analysis and its interpretation appropriate?

Yes

Are all the source data underlying the results available to ensure full reproducibility?

Yes

Are the conclusions drawn adequately supported by the results?

Yes

Are sufficient details of methods and analysis provided to allow replication by others?

Yes

Reviewer Expertise:

Microbiology, Epidemiology, Microbiomes

I confirm that I have read this submission and believe that I have an appropriate level of expertise to confirm that it is of an acceptable scientific standard.

# Reviewer report: Reviewer response for version 2

**DOI:** 10.21956/openreseurope.26461.r77754

**Published:** 2026-07-24

**Authors:** Thang Nguyen

**Affiliations:** 1 Can Tho University of Medicine and Pharmacy, Can Tho, Vietnam

Thank you for the substantial revision. The manuscript is clearer, and several previous concerns have been addressed. However, a number of important issues remain that affect the reproducibility and interpretation of the study.

**1. Individual preparedness remains insufficiently reported.**

 The Abstract, Discussion, and Conclusions state that healthcare workers demonstrated good or relatively high self-reported individual preparedness. However, the Methods do not define the individual-preparedness instrument, number of items, scoring procedure, possible score range, classification threshold, or reliability, and the Results contain no corresponding table or numerical estimate. Please either report this outcome fully, including the score distribution and numerator/denominator for each category, or remove all claims regarding good individual preparedness. Item-level findings discussed in the Discussion, such as recognition of the case definition and outbreak threshold, should also be presented in the Results. In addition, the conceptual framework should be described as hypothetical because the proposed pathways from knowledge to IPC practice and preparedness were not tested in the reported analyses.

**2. The sampling structure and clustering require further attention.**

 Participants were nested within facilities and districts, yet the logistic regression models do not appear to account for within-facility correlation. Moreover, the tertiary-care participants appear to have been recruited from a single purposively selected tertiary facility. Consequently, the strong association between facility level and IPC practice may partly represent a single-facility effect rather than a general difference between levels of care. Please specify the number and type of facilities contributing healthcare-worker participants, clarify whether these were the same 24 facilities included in the facility assessment, and report the numbers approached, refusing participation, and excluded because of missing data. Cluster-robust or multilevel analyses should be considered where methodologically feasible. Otherwise, the inference should be substantially tempered and this limitation explicitly acknowledged. Generalizability should also be restricted to the sampled nurses and community health extension workers in selected public facilities and LGAs, rather than all healthcare workers in Kano State.

**3. The outcome classifications require clearer justification.**

 The use of the sample median to classify knowledge and IPC practice almost inevitably divides respondents into approximately equal groups. Therefore, the terms “poor” and “good” imply a criterion-referenced standard that has not been demonstrated. Please report the underlying continuous score distributions and either use neutral terms such as “lower” and “higher” scores or provide a validated external basis for the selected thresholds. Item scoring, treatment of missing responses, and any reverse-coded items should also be specified. Facility preparedness is described as a 33-item measure, whereas Table 6 presents only 22 items. The complete checklist and scoring guide should be provided, and the authors should clarify whether the 0–11, 12–22, and 23–33 categories are prespecified absolute score bands, tertiles, or percentile-based cut-offs.

**4. Several numerical and statistical inconsistencies remain.**

 Please undertake another complete verification against the statistical output. In particular:

- In Table 4, a coefficient of −0.638 corresponds to an AOR of approximately 0.528, not 0.428; the reported confidence interval of 0.322–0.868 is also consistent with an estimate of approximately 0.53.
- The displayed collapsed marital-status counts in Table 2 do not appear to support χ² = 6.779 and p = 0.028. Please recalculate the test and specify whether Pearson’s chi-square or Fisher’s exact test was used.
- In Table 3, the certificate/diploma counts sum to 202, although Table 1 reports 201 participants in this category.
- The p-value for Kano South is missing from Table 5.
- In Table 6, 14/24 is 58.3%, not 56.3%; 15/24 is 62.5%, not 62.6%; and 5/24 is 20.8%, not 8.2%.
- The 216 respondents shown in Figure 2 represent 51.3% of 421, rather than 51.0%.

Adjusted results should consistently be labelled as AORs, and interpretations should refer to “lower odds” rather than “less likelihood,” particularly because these are common cross-sectional outcomes.

**5. Reproducibility and presentation still need improvement.**

 The author response states that facility-level clustering was acknowledged, but this limitation is not included in the Strengths and Limitations section. The data availability statement still does not identify a codebook, complete scoring guide, or analysis syntax; these materials should be deposited to permit reproducibility. A completed STROBE checklist would also be helpful. The software statement should be corrected because the statement that all software was open access conflicts with the subsequent statement that SPSS was licensed; “SPSS version 20iiii” and the unnecessary serial number should also be removed. In Table 6, treatment readiness should refer to prompt treatment of suspected or clinically diagnosed cases rather than waiting for confirmation, in accordance with the clinical guidance cited elsewhere in the manuscript. A final technical language edit remains necessary, including correction of expressions such as “internal health regulations,” “staffs,” the duplicate funder statement, and residual grammatical errors in the Abstract and tables.

 The study addresses an important public-health problem and the revision represents meaningful progress. Nevertheless, the unresolved outcome-definition, sampling, clustering, scoring, and numerical issues remain sufficiently important that I retain the status **Approved with Reservations**.

Is the study design appropriate and does the work have academic merit?

Yes

Is the work clearly and accurately presented and does it cite the current literature?

Partly

If applicable, is the statistical analysis and its interpretation appropriate?

Partly

Are all the source data underlying the results available to ensure full reproducibility?

Partly

Are the conclusions drawn adequately supported by the results?

Partly

Are sufficient details of methods and analysis provided to allow replication by others?

No

Reviewer Expertise:

PharmacoTherapy and Pharmaceutical Care

I confirm that I have read this submission and believe that I have an appropriate level of expertise to confirm that it is of an acceptable scientific standard, however I have significant reservations, as outlined above.

# Response

**Published:** 2026-08-28

**Authors:** Feyisara Ayodele

**Affiliations:** Epidemiology and Medical Statistics, University of Ibadan Faculty of Public Health, Ibadan, Oyo, Nigeria

1. Thank you for the comment. We have removed unsupported claims regarding individual preparedness and revised the manuscript accordingly. We have also ensured that item-level findings are reported in the Results and clarified that the pathways in the conceptual framework are hypothetical and were not tested in this study.

 2. Thank you for the comment. We have clarified the participating facilities, recruitment and exclusion numbers, and their relationship with the 24 assessed facilities. Given the facility structure and single tertiary-facility recruitment, we have tempered the interpretation of facility-level associations, explicitly acknowledged the potential clustering and single-facility effect as limitations, and restricted generalizability to the sampled nurses and CHEWs in selected public facilities and LGAs.

 3. Thank you for the comment. We have clarified the scoring procedures and handling of missing and reverse-coded items and provided the complete checklist and scoring guide. We have also clarified the basis of the preparedness categories and revised the terminology for knowledge and IPC practice to avoid implying validated criterion-based thresholds.

 4. Thank you for the careful review. We have rechecked the statistical outputs and corrected all identified numerical and statistical inconsistencies across all tables.

 5. Thank you for the comments. We have addressed all the issues raised, including adding the clustering limitation and scoring guide, correcting the software statement, revising Table 6, and proofreading the manuscript to correct terminology, duplication, and grammatical errors.

# Reviewer report: Reviewer response for version 1

**DOI:** 10.21956/openreseurope.24019.r73969

**Published:** 2026-05-29

**Authors:** Thang Nguyen

**Affiliations:** 1 Can Tho University of Medicine and Pharmacy, Can Tho, Vietnam

The manuscript addresses an important and timely public health topic: preparedness for diphtheria outbreaks among healthcare workers in Kano State, Nigeria. The study uses a descriptive cross-sectional survey of nurses and community health extension workers and reports substantial gaps in diphtheria knowledge, infection prevention and control practices, and facility-level preparedness. The topic has clear academic and policy merit, particularly given the recent diphtheria resurgence in Nigeria and the critical role of frontline healthcare workers in outbreak detection, IPC, treatment initiation, vaccination counseling, and referral.

 Overall, the study has potential value, but I have significant reservations about the current version. The main findings are relevant, but the manuscript requires substantial revision of the methods, statistical reporting, interpretation, and discussion before the work can be considered scientifically sound and reproducible.

**Major points that must be addressed**

1. **Clarify the conceptual framework and align the title, aims, results, and conclusions.**
  The title focuses on “preparedness,” but the manuscript presents knowledge, IPC practice, individual preparedness, and facility preparedness as separate or overlapping constructs. The authors should clearly define “preparedness” and explain whether knowledge and IPC practice are components of preparedness or independent outcomes. A brief conceptual framework would help. The abstract and conclusion should then align with this framework.
2. **The methods section is too brief to allow replication.**
  The authors should provide sufficient detail for another researcher to reproduce the study. Methods should include: the study setting and the number and type of facilities sampled; the sampling frame; detailed steps of the two-stage or multistage sampling procedure; sample size calculation and assumptions; inclusion and exclusion criteria; recruitment procedure; response rate; dates of data collection; interviewer training; whether the questionnaire was developed, adapted, or adopted;  the language and translation process; pilot testing; validity and reliability assessment; and handling of missing data.
3. **Scoring systems must be fully described.**
  The manuscript reports “poor” versus “good” knowledge, “poor” versus “good” IPC practice, and “poor/moderate/good” facility preparedness, but the scoring rules are insufficiently described. The authors should specify the number of items in each domain, the scoring for each item, the total possible score, the cut-offs used to classify poor/good or poor/moderate/good, and the justification for these cut-offs. Without this information, the main outcomes cannot be replicated or interpreted with confidence.
4. **Facility preparedness assessment needs clearer reporting.** Table 6 appears to use a facility-level denominator of 24, but the manuscript does not clearly describe how the facilities were selected, who completed the checklist, whether the checklist was based on direct self-report, or how the overall facility preparedness category was derived. This is especially important because the conclusion highlights poor facility preparedness as a major finding. 
5. **Statistical analysis and interpretation require revision. **Chi-square tests and logistic regression are generally appropriate for the stated outcomes, but the analysis plan is under-described. The authors should specify outcome coding, reference categories, variables included in each model, the rationale for model selection, how sparse categories were handled, and whether clustering by facility or district was considered. Some cells are small, for example, the “others” ethnicity and divorced/separated/widowed marital status categories, so the authors should consider Fisher’s exact test or collapsing categories where appropriate.
  The authors should also clearly distinguish between bivariate associations and multivariable predictors. For example, the manuscript reports several bivariate associations, but many variables are not statistically significant in the multivariable models. Non-significant variables should not be described as “predictors.” The wording in the abstract and conclusion should be revised accordingly.
6. **Check and correct numerical and table inconsistencies. **Several numerical or reporting issues need attention. For example, the text reports female respondents as 54.7%, whereas Table 1 gives 228/421 = 54.2%. The mean age is written as 34(9.2%) years, but the standard deviation should not be expressed as a percentage. In Table 2, the percentage for Igbo respondents with good knowledge appears inconsistent with the denominator. Some p-values appear missing, such as for marital status in Table 2 and Kano South in Table 5. These should be corrected.
7. **Update and strengthen the literature review.**
  The introduction is relevant but should cite more current, disease-specific literature and guidelines. The authors should incorporate recent guidance on diphtheria clinical management, IPC/WASH measures, and healthcare personnel exposure management. WHO lists 2024 materials on clinical management, IPC/WASH, and diphtheria antitoxin safety, and its diphtheria page also summarizes vaccination, antibiotics, antitoxin, and early treatment principles. CDC’s 2024 healthcare personnel guidance includes recommendations on post-exposure prophylaxis, work exclusion, cultures, monitoring, vaccination, and droplet/contact precautions. The authors should also add recent Kano/Nigeria outbreak literature, such as the 2025 EBioMedicine analysis of 18,320 Kano cases and the 2025 Infectious Disease Modelling paper on transmission dynamics in Kano. 
8. **Improve the discussion.**
  The discussion currently repeats many descriptive results rather than interpreting them. The authors should restructure it around the main findings: knowledge gaps, IPC practice gaps, primary facility vulnerabilities, and poor facility preparedness. They should compare their findings with disease-specific diphtheria literature and outbreak preparedness literature, explain plausible reasons for the findings, and discuss implications for training, availability of PPE, isolation capacity, antitoxin/antibiotic readiness, surveillance, vaccination systems, and primary healthcare strengthening.
9. **Revise the conclusion so that it is directly supported by the results.**
  The conclusions that HCWs had poor knowledge and poor IPC practice, and that facility preparedness was poor, are generally supported. However, the conclusion should not list variables as “key predictors” unless they were statistically significant in the multivariable models. The statement about political instability and logistic factors should be removed unless these were measured and presented in the results. The statement that 78.1% of respondents demonstrated good preparedness should be supported by a clearly presented table or analysis in the results section.
10. **Language and presentation need professional editing.**
  The manuscript contains grammar, punctuation, and terminology problems that affect clarity. Examples include “this review aims” despite the study being original research, “staffs”, “not loose guards”, and the phrase “low vaccine coverage of diphtheria antitoxin,” which is inaccurate because antitoxin is a treatment, not a vaccine. The manuscript should undergo language editing and careful technical review.

**Minor comments**

 The authors should report the study according to the STROBE checklist for cross-sectional studies. They should also ensure consistency in the software description, as the methods mention SPSS version 21, whereas the software availability statement mentions SPSS version 20 and Stata version 12.1. If Stata was used, the analyses performed in Stata should be specified; if not, Stata should be removed. The data availability statement is useful, but reproducibility would be improved by providing a codebook, scoring guide, and analysis syntax.

**Overall assessment**

 This manuscript addresses an important topic and the cross-sectional design is acceptable for assessing knowledge, IPC practice, and preparedness at a specific time point. However, the current manuscript is not sufficiently detailed for replication, and some statistical interpretations and conclusions require correction. I recommend approval with reservations, conditional on substantial revision of the methods, scoring procedures, statistical reporting, discussion, and conclusions.

Is the study design appropriate and does the work have academic merit?

Yes

Is the work clearly and accurately presented and does it cite the current literature?

Partly

If applicable, is the statistical analysis and its interpretation appropriate?

Partly

Are all the source data underlying the results available to ensure full reproducibility?

Partly

Are the conclusions drawn adequately supported by the results?

Partly

Are sufficient details of methods and analysis provided to allow replication by others?

No

Reviewer Expertise:

PharmacoTherapy and Pharmaceutical Care

I confirm that I have read this submission and believe that I have an appropriate level of expertise to confirm that it is of an acceptable scientific standard, however I have significant reservations, as outlined above.

# Response

**Published:** 2026-06-26

**Authors:** Feyisara Ayodele

**Affiliations:** Epidemiology and Medical Statistics, University of Ibadan Faculty of Public Health, Ibadan, Oyo, Nigeria

1. Thank you for this observation. We have added a conceptual framework and revised the manuscript to clearly define preparedness and distinguish knowledge and IPC practices as determinants of preparedness.

 2. Thank you for this observation. We have revised the Methods section to provide sufficient detail on the study design, sampling, data collection, questionnaire development, and statistical analysis to improve transparency and reproducibility.

 3. Thank you for this observation. We have revised the Methods section to clearly describe the scoring procedures, including the scoring criteria, classification thresholds, and justification for the selected cut-off points.

 4. Thank you for this observation. We have revised the Methods section to clearly describe the facility preparedness assessment, including facility selection, checklist administration, data sources, and the derivation of facility preparedness scores.

 5. Thank you for this comment. We have revised the Statistical Analysis section to clarify the analytical approach, handling of sparse data, and interpretation of multivariable results and acknowledged facility-level clustering as a study limitation.

 6. Thank you for this observation. We have reviewed and corrected all tables, percentages, descriptive statistics, and p-values and cross-checked them against the text to ensure accuracy and consistency throughout the manuscript.

 7. Thank you for this valuable suggestion. We have updated the introduction with recent WHO and CDC guidance and current evidence from the Nigerian diphtheria outbreak to provide a stronger, disease-specific rationale for the study.

 8. Thank you for this comment. We have revised the Discussion to emphasize interpretation, comparison with relevant literature, and the implications for policy and practice.

 9. Thank you for this observation. We have revised the Conclusion to ensure it reflects only the study findings, removing unsupported statements and restricting conclusions to variables that remained significant after multivariable analysis.

 10. Thank you for this observation. The manuscript has been thoroughly edited to improve grammar, clarity, consistency, and scientific terminology, and the identified language issues have been corrected throughout.

# Reviewer report: Reviewer response for version 1

**DOI:** 10.21956/openreseurope.24019.r73963

**Published:** 2026-05-29

**Authors:** David Broderick

**Affiliations:** 1 The University of Auckland, Auckland, New Zealand

This paper represents an important aspect of epidemic preparedness- in assessing baseline knowledge prior to an existing epidemic.

 The methodology of the paper seems sound, however there are aspects of the manuscript which could be expanded further to better highlight the key elements of the study and explain some of the authors choices. I have made a detailed list below:

 1. The authors use several demographic variables in their reporting in particular (marriage status, religion and ethnic group) but don't introduce what these groups are and why they might need to be considered and important. This is relevant as the results are presented with this lens in mind, but I'm unsure differences within these groups would be common knowledge amongst the readership

 2. Similarly while the results are split by demographics, there is currently insufficient discussion of the meaning as to why these knowledge disparities may exist- and more importantly- what can be done about them.

 3. Figure 1 should be represented as a plain 2D figure as 3D can be difficult to interpret and mislead readers

Is the study design appropriate and does the work have academic merit?

Yes

Is the work clearly and accurately presented and does it cite the current literature?

Yes

If applicable, is the statistical analysis and its interpretation appropriate?

Yes

Are all the source data underlying the results available to ensure full reproducibility?

Yes

Are the conclusions drawn adequately supported by the results?

Yes

Are sufficient details of methods and analysis provided to allow replication by others?

Yes

Reviewer Expertise:

Microbiology, Epidemiology, Microbiomes

I confirm that I have read this submission and believe that I have an appropriate level of expertise to confirm that it is of an acceptable scientific standard, however I have significant reservations, as outlined above.

# Response

**Published:** 2026-06-26

**Authors:** Feyisara Ayodele

**Affiliations:** Epidemiology and Medical Statistics, University of Ibadan Faculty of Public Health, Ibadan, Oyo, Nigeria

1. Thank you for this observation. We have revised the manuscript to clarify the relevance of marital status, religion, and ethnicity by explaining their potential influence on health knowledge, risk perception, healthcare utilisation, and public health behaviours. We also added brief descriptions of these variables to improve understanding for readers unfamiliar with the Nigerian context. 2. Thank you for this valuable observation. We have revised the Discussion to explain the observed socio-demographic differences using relevant literature and highlighted their public health implications, including targeted health education, risk communication, and healthcare worker training. 3. Thank you for this suggestion. Figure 1 has been revised to a two-dimensional format to improve clarity, readability, and interpretation of the results.

# Reviewer report: Reviewer response for version 1

**DOI:** 10.21956/openreseurope.24019.r73369

**Published:** 2026-05-12

**Authors:** Ukamaka Gladys Okafor

**Affiliations:** 1 Euclid University, Bangui, Central African Republic

The paper's title focuses on preparedness, and the abstract and body of the manuscript include this knowledge. Are the authors assuming that knowledge is part of preparedness?

 2. The last sentence in the introduction section described the study as a review, but the study is original research.

 3. Authors are advised to subject the manuscript to a language check, including punctuation.

 4. The method section was too brief and vague, and did not explain the study design and procedure for sampling and data collection, including the source of the survey tool, whether it was developed, adapted, or adopted. How and where did you recruit the participants

 5. The discussion section was visibly a repetition of results, including figures. The conclusion also contains some results figures. The discussion is expected to analyze the findings, compare them with the literature review, explain how they relate to current practice, and discuss the policy implications.

 Thank you

Is the study design appropriate and does the work have academic merit?

Yes

Is the work clearly and accurately presented and does it cite the current literature?

Yes

If applicable, is the statistical analysis and its interpretation appropriate?

Yes

Are all the source data underlying the results available to ensure full reproducibility?

Yes

Are the conclusions drawn adequately supported by the results?

Yes

Are sufficient details of methods and analysis provided to allow replication by others?

No

Reviewer Expertise:

Public Health, Policy and regulation,

I confirm that I have read this submission and believe that I have an appropriate level of expertise to confirm that it is of an acceptable scientific standard, however I have significant reservations, as outlined above.

# Response

**Published:** 2026-06-26

**Authors:** Feyisara Ayodele

**Affiliations:** Epidemiology and Medical Statistics, University of Ibadan Faculty of Public Health, Ibadan, Oyo, Nigeria

1. Thank you for this valuable observation. We have revised the manuscript to clarify that preparedness is a multidimensional construct comprising knowledge, infection prevention and control (IPC) practices, and individual and facility readiness for diphtheria outbreak response. Knowledge is presented as a foundational determinant of preparedness rather than a standalone outcome.

 We have also added a conceptual framework and revised the Introduction and Methods sections to clearly distinguish and explain the relationships between knowledge, IPC practices, and preparedness

 2. Thank you for identifying this oversight. We have corrected the final paragraph of the Introduction to accurately reflect the study design and objectives.

 The revised sentence now states: *"This study aimed to assess the knowledge, infection prevention and control practices, and preparedness of healthcare workers for diphtheria outbreak response in Kano State, Nigeria."*

*3. *Thank you for this valuable suggestion. The manuscript has been thoroughly edited and proofread to improve grammar, punctuation, clarity, and overall readability, ensuring consistency with the journal's language requirements.

 4. Thank you for this important observation. We have substantially revised the Methods section to provide a clearer and more comprehensive description of the study design, setting, participant selection, sampling and recruitment procedures, data collection methods, survey instrument development, validity and reliability assessment, and statistical analysis, thereby improving the transparency, reproducibility, and clarity of the study.

 5. Thank you for this valuable comment. We have revised the Discussion to emphasize interpretation, comparison with relevant literature, and the implications for practice and policy, rather than repeating the results. The conclusion has also been revised to remove numerical findings and provide a concise summary of the study's key implications and recommendations.
